# Anatomical Risk Factors for Patellofemoral Instability Vary by Dejour Type of Trochlear Dysplasia

**DOI:** 10.1002/ars2.70123

**Published:** 2026-11-15

**Authors:** Martin Hartmann, Berfin Caliskan, Lazaros Vlachopoulos, Jakob Ackermann, Sandro F. Fucentese

**Affiliations:** ^1^ Department of Orthopedics and Trauma Surgery Lucerne Cantonal Hospital Luzern Switzerland; ^2^ Department of Orthopedics Balgrist University Hospital, University of Zurich Zurich Switzerland; ^3^ Department of Orthopedic Surgery Midwest Orthopaedics at Rush Chicago Illinois U.S.A.

## Abstract

**Purpose:**

To assess whether Dejour trochlear dysplasia (TD) types differ in their association with other patellofemoral anatomical variables in patients with patellar instability.

**Methods:**

All patients with TD undergoing evaluation for patellar instability between January 2010 and December 2020 were included if a true lateral radiograph and a preoperative magnetic resonance imaging were available. Two reviewers independently assessed TD type (Dejour classification), femorotibial rotation, tibial tubercle‐trochlear groove (TTTG) distance, sagittal TTTG, tibial tubercle‐to‐posterior cruciate ligament distance, supratrochlear spur height, sulcus depth, Merchant's congruence angle, trochlear sulcus angle, Caton‐Deschamps index, patellotrochlear index, patellar tilt, angle, thickness, and width.

**Results:**

The study included 330 patients (363 knees, 66.7% women, mean age 22.5 ± 7.9 years, body mass index 24.9 ± 5.4). TD distribution was type A (17.1%), B (33.6%), C (25.9%), and D (23.4%). Types B and D showed significantly increased Merchant's congruence angle (*P* = .019), patellar tilt (*P* < .001), patellotrochlear index (*P* = .035), TTTG (*P* < .001), sagittal TTTG (*P* < .001), and femorotibial rotation (*P* < .001).

**Conclusions:**

Patients with Dejour type B and D TD had significantly increased values in other patellofemoral instability risk factors compared with those presenting with type A and C dysplasia.

**Level of Evidence:**

Level IV, diagnostic retrospective case series.

Trochlear dysplasia (TD) is frequently observed in patients with patellar instability and refers to an abnormal formation of the trochlear groove, which is a major risk factor for patellofemoral (PF) instability.^1^ Because of inadequate patellar containment within the trochlea, this condition may lead to recurrent patellar dislocations.^1^

The Dejour classification is the most widely used system for categorizing TD based on radiographic and magnetic resonance imaging (MRI) findings. It distinguishes 4 types:^2^

• Type A: a shallow trochlea with a crossing sign indicating trochlear flattening, without a supratrochlear spur and without a double contour.

• Type B: a flat or convex trochlea characterized by the presence of a supratrochlear spur but without a double contour.

• Type C: a lateral convexity with medial hypoplasia, presenting as a double contour on imaging, without a supratrochlear spur.

• Type D: a severe dysplastic morphology characterized by a supratrochlear spur, a double contour indicating medial facet hypoplasia, and the “cliff” sign, which represents a sharp transition between the medial and lateral facets.

This classification helps assess the severity of TD and guides treatment decisions, particularly in cases of recurrent patellar dislocation.^2^ TD significantly impacts PF biomechanics, leading to increased internal rotation, lateral tilt, and lateral translation of the patella.^3^ Additionally, it results in elevated contact pressures, reduced contact area, and decreased joint stability.^4^, ^5^, ^6^ TD types B and D may exert a particularly pronounced influence on PF biomechanics. The presence of a supratrochlear spur has been associated with increased PF compression during knee flexion, which in turn may contribute to cartilage degeneration and the development of isolated PF osteoarthritis.^3^, ^7^ Therefore, surgical correction of TD is currently recommended particularly in patients with Dejour types B and D.^8^, ^9^, ^10^

TD often coexists with other anatomical abnormalities that contribute to patellar instability. For instance, patella alta, characterized by an elevated patellar height, is frequently observed in patients with TD.^11^

Genu valgum can increase lateralizing forces on the patella and is another risk factor that often accompanies TD.^12^ A lateralized tibial tubercle, measured by an increased tibial tubercle‐trochlear groove (TTTG) distance, is significantly associated with TD and contributes to patellar instability.^13^ Recently, the sagittal TTTG (sTTTG) distance has also been identified as an additional risk factor that is associated with TD.^13^ Lastly, excessive lateral patellar tilt is frequently observed in patients with TD, leading to increased lateral patellar tracking and instability.^14^, ^15^ These anatomical risk factors, along with various others, interact in a complex manner, increasing the likelihood of patellar instability and influencing the clinical management of these patients. Although previous studies reported that TD is generally associated with other common PF anatomical risk factors, only limited data exist regarding the differences seen when stratified by dysplasia severity according to Dejour.^6^, ^13^, ^15^, ^16^, ^17^, ^18^

The purpose of this study was therefore to assess whether Dejour TD types differ in their association with other PF anatomical variables in patients with patellar instability. We hypothesized that high‐grade TD (types B and D) would show stronger associations with other PF anatomical risk factors compared with low‐grade TD (types A and C).

## METHODS

Ethical approval was granted by the local research ethics committee, and all included patients gave their written consent.

### Patient Selection

Patients evaluated for PF instability at a single academic institution between January 2010 and December 2020 were screened for eligibility. Inclusion criteria required a preoperative lateral radiograph and MRI of the affected knee. The diagnosis of TD was confirmed by a musculoskeletal radiologist and an orthopaedic surgeon (J.A.) based on the lateral radiograph and preoperative MRI. Patients with a history of prior PF surgical stabilization on the affected knee were excluded.

### Clinical and Radiographic Assessment

Clinical notes and operative reports were reviewed to determine the patient's age at the time of surgery, biological sex, and body mass index (BMI). Sex was defined as the biological classification (female or male) as recorded in the medical record at the time of initial presentation. To comply with the sex and gender equity in research recommendations, all demographic and anatomical variables were additionally compared between female and male patients using sex‐stratified analyses. In addition to patient characteristics and TD type according to Dejour, 2 independent reviewers (M.H., B.C., orthopaedic residents) assessed femorotibial (FT) rotation, TTTG distance, sTTTG distance, tibial tubercle‐to‐posterior cruciate ligament (TT‐PCL) distance, supratrochlear spur height, sulcus depth, Merchant's congruence angle, trochlear sulcus angle, patellotrochlear index (PTI), patellar tilt, patellar angle, patellar thickness, patellar width, and Caton‐Deschamps index (CDI). Both readers were experienced in performing the respective measurements, were blinded to clinical data and each other's results, and had received extensive training in the measurement protocol prior to the study. Additionally, both had previously participated in related research projects involving similar measurement analyses.

FT rotation (equivalent to knee rotation) was measured on axial images between the tangent of the posterior femoral condyles and the tangent of the posterior tibial plateau.^19^, ^20^ A positive angle indicated external rotation of the tibia relative to the femur.^19^ TTTG was assessed between the most anterior point of the tibial tuberosity and the deepest point of the trochlear groove, perpendicular to the tangent to the bony borders of the posterior condyles on axial scans. A perpendicular line through the deepest point of the bony trochlear groove defined the trochlear reference. The tibial tubercle was identified at the most cephalad axial slice showing full patellar tendon contact, with its center marked at the midpoint of the tendon insertion.^21^

sTTTG distance was measured on axial images and is defined as the sagittal and not coronal distance between a point at the nadir of the trochlear cartilage and the most anterior point of the tibial tubercle at the attachment of the patellar tendon, which is referenced to the posterior condylar axis for orientation.^13^, ^22^ The TT‐PCL was identified on axial imaging and defined on the most inferior slice on which the ligament could be clearly visualized. The dorsal tibial condylar line was established on the axial slice immediately distal to the articular surface of the tibial plateau and proximal to the fibular head. The TT‐PCL distance was defined as the mediolateral distance between the midpoint of the tibial tubercle and the medial border of the PCL. Measurements were performed parallel to the dorsal tibial condylar line.^23^ The supratrochlear spur height is defined as the maximum distance between a tangent drawn along the anterior cortex of the distal femur and a parallel line passing through the tip of the supratrochlear spur.^24^

Trochlear sulcus depth is measured as the vertical distance from the deepest point of the trochlear groove to a line connecting the highest points of the medial and lateral femoral condyles.^13^ Merchant's congruence angle characterizes the alignment of the patella within the trochlear groove and is defined by the intersection of a line that bisects the sulcus angle and a line connecting the deepest point of the trochlea to the most posterior point along the articular ridge of the patella.^25^ The sulcus angle was measured on the axial images showing the greatest mediolateral distance between the femoral epicondyles. All measurements were based exclusively on osseous landmarks. The angle was defined by lines drawn from the deepest osseous point of the trochlear groove to the most anterior osseous margins of the medial and lateral trochlear facets.^26^ The PTI is a radiographic measurement used to assess the relation between the patella and the trochlea. The PTI is measured on sagittal MRI. It is calculated as the ratio of the length of the trochlea that is overlapped by the patella divided by the total length of the patellar articular cartilage. This ratio is expressed as a percentage. A normal PTI value is around 31.7% in healthy individuals without PF complaints.^27^ Patellar tilt is measured on axial MR images between a line drawn along the widest part of the patella and a line parallel to the posterior condyles of the femur.^28^ The patellar angle is defined as the angle between a line drawn along the lateral facet of the patella and a line parallel to the posterior condyles of the femur in the axial plane at the level of the greatest transverse diameter of the patella.^29^ Patellar thickness is measured at its thickest part, which is usually at the center of the patella in the axial view.^30^ Patellar width measurement is taken at the widest part of the patella on axial images.^30^, ^31^ CDI is calculated as the ratio of the distance from the most inferior margin of the patellar articular cartilage to the anterosuperior aspect of the tibial plateau to the length of the patellar articular cartilage.^32^

All reported parameters were measured on the MRI images. The MRI examinations were performed at the same center using standardized patient positioning and identical coils to ensure the comparability of measurements. Imaging was conducted with either a 1.5‐T MAGNETOM Avanto Fit or a 3.0‐T MAGNETOM Verio system (Siemens Medical Solutions, Erlangen, Germany), applying a dedicated musculoskeletal protocol with coronal, sagittal, and axial sequences at a slice thickness of 3 mm. All images were read independently on a picture archiving and communication system workstation certified for clinical use (MERLIN 7.1.22, Phönix‐PACS GmbH). In the event of disagreement, a third observer (J.A., a board‐certified orthopaedic surgeon) assessed the images to achieve consensus.

### Statistical Analysis

Sociodemographic and clinical characteristics of the study population were analyzed using descriptive statistics. Continuous variables are expressed as mean ± standard deviation. The normality of data distribution was assessed using the Shapiro‐Wilk test. Depending on the distribution characteristics, appropriate parametric or nonparametric tests were applied to evaluate differences in anatomical parameters between the 4 Dejour types of TD. All statistical analyses were conducted using SPSS for Mac (Version 23.0, SPSS, Chicago, IL), with statistical significance defined as *P* < .05.

## RESULTS

The study cohort included 363 knees from 330 patients (66.7% women), with an average age of 22.5 ± 7.9 years and a mean BMI of 24.9 ± 5.4. Among the included knees, 17.1% presented with Dejour type A, 33.6% with type B, 25.9% with type C, and 23.4% with type D. When stratified by Dejour classification, no statistically significant differences were found in age, sex, BMI, patellar width, patellar angle, patellar thickness, CDI, or TT‐PCL (all *P* > .05).

In contrast, several parameters reflecting PF alignment and trochlear morphology showed significant overall group differences (Table 1). Increasing dysplasia severity was associated with progressive alterations in both axial alignment and trochlear anatomy. Specifically, patellar tilt, PTI, trochlear sulcus angle, supratrochlear spur height, TTTG distance, sTTTG distance, Merchant's congruence angle, and FT rotation differed significantly among groups, whereas sulcus depth showed a corresponding decrease with higher dysplasia grades. Sex‐stratified analyses revealed no significant differences in the distribution of Dejour TD types between female and male patients (*P* = .864). Likewise, no significant differences were observed for age, BMI, CDI, PTI, trochlear sulcus angle, Merchant's congruence angle, sulcus depth, trochlear spur height, TTTG distance, FT rotation, patella alta, Wiberg classification, or history of patellar dislocation (all *P* > .05). Female patients had significantly greater patellar tilt (29.3 ± 10.7° vs 25.0 ± 10.8°, *P* = .001) and sTTTG distance (9.9 ± 5.2 vs 8.4 ± 6.0 mm, *P* = .005), whereas male patients had significantly greater patellar width (41.3 ± 4.1 vs 37.9 ± 3.4 mm, *P* < .001), patellar thickness (18.5 ± 2.6 vs 16.8 ± 2.0 mm, *P* < .001), and TT‐PCL distance (20.7 ± 4.0 vs 19.2 ± 3.5 mm, *P* = .001).

**TABLE 1:** Comparison of Demographic Characteristics and Anatomical Parameters Across Dejour Types A‐D

**Parameter**	**Dejour A (n = 62; 17.1%)**	**Dejour B (n = 122; 33.6%)**	**Dejour C (n = 94; 25.9%)**	**Dejour D (n = 85; 23.4%)**	***P*** **Value**
Age, yr	22.2 ± 7.1	21.7 ± 7.3	23.2 ± 8.7	23.2 ± 8.4	.66
BMI	24.9 ± 5.3	25.2 ± 5.6	24.4 ± 5.6	25.0 ± 5.1	.539
Patellar width, mm	39.8 ± 3.5	39.4 ± 3.8	38.5 ± 4.4	39.5 ± 3.7	.178
Patellar thickness, mm	17.6 ± 2.5	17.6 ± 2.3	17.3 ± 2.5	17.4 ± 2.3	.756
Patellar angle, °	123.2 ± 11.9	125.4 ± 11.8	122.6 ± 14.0	126.4 ± 12.8	.287
Patellar tilt, °	20.0 ± 8.4	28.8 ± 12.0	23.3 ± 10.1	32.9 ± 12.3	<.001
CDI	1.2 ± 0.2	1.2 ± 0.2	1.2 ± 0.2	1.2 ± 0.2	.114
PTI, %	38 ± 14	41 ± 14	38 ± 15	44 ± 18	.035
Trochlear sulcus angle, °	156.8 ± 6.3	162.1 ± 7.0	160.7 ± 7.5	163.7 ± 7.1	<.001
Merchant's congruence angle, °	34.4 ± 26.3	40.3 ± 28.3	34.5 ± 22.7	45.6 ± 32.6	.019
Sulcus depth, mm	2.4 ± 0.8	2.0 ± 0.8	2.1 ± 1.0	1.8 ± 0.8	<.001
Trochlear spur height, mm	4.2 ± 0.9	5.4 ± 1.6	4.7 ± 1.3	5.8 ± 1.8	<.001
TTTG distance, mm	12.4 ± 4.7	13.9 ± 4.3	13.9 ± 4.1	16.8 ± 6.0	<.001
TT‐PCL distance, mm	18.9 ± 4.0	19.8 ± 4.0	19.5 ± 3.1	20.0 ± 4.0	.141
sTTTG distance, mm	6.5 ± 5.1	10.0 ± 5.4	7.6 ± 4.9	10.5 ± 5.9	<.001
FT rotation, °	1.3 ± 7.7	5.1 ± 6.8	3.0 ± 7.5	7.7 ± 7.3	<.001

*Note*: *P* value indicates whether there is a statistically significant difference among the 4 types.

BMI, body mass index; CDI, Caton‐Deschamps index; FT, femorotibial; PTI, patellotrochlear index; sTTTG, sagittal tibial tubercle‐trochlear groove; TT‐PCL, tibial tubercle‐to‐posterior cruciate ligament; TTTG, tibial tubercle‐trochlear groove; yr, year.

Pairwise comparisons revealed a consistent gradient across dysplasia severity (Table 2). Type D showed significantly greater patellar tilt than all other subtypes, whereas type A exhibited the lowest values. Types B and D both showed significantly increased patellar tilt than types A and C. PTI values were significantly higher in type D than in lower‐grade dysplasia types, and type B also differed from type C.

**TABLE 2:** Pairwise Comparison

**Parameter**	**A vs B**	**A vs C**	**A vs D**	**B vs C**	**B vs D**	**B vs D**
Patellar tilt, °	<0.001	0.031	<0.001	<0.001	0.013	<0.001
PTI	0.167	0.739	0.047	0.049	0.41	0.001
Trochlear sulcus angle, °	<0.001	<0.001	<0.001	0.257	0.083	0.007
Merchant's congruence angle, °	0.116	0.845	0.01	0.12	0.186	0.072
Sulcus depth, mm	0.001	0.008	<0.001	0.473	0.226	0.72
Trochlear spur height, mm	<0.001	0.065	<0.001	<0.001	0.17	<0.001
TTTG distance, mm	0.27	0.4	<0.001	0.949	<0.001	<0.001
sTTTG distance, mm	<0.001	0.365	<0.001	<0.001	0.719	<0.001
FT rotation	0.005	0.519	<0.001	0.137	0.057	<0.001

*Note*: *P* value indicates whether there is a statistically significant difference.

FT, femorotibial; PTI, patellotrochlear index; sTTTG, sagittal tibial tubercle‐trochlear groove; TTTG, tibial tubercle‐trochlear groove.

Trochlear sulcus angle increased progressively with dysplasia severity. Type A differed significantly from all higher‐grade subtypes, and additional differences were observed between type C and type D. Merchant's congruence angle showed significant differences primarily between type D and the lower‐grade subtypes, with type D showing the greatest values. Sulcus depth showed an inverse pattern, with type A differing significantly from the other subtypes and exhibiting the greatest depth, whereas type D showed the smallest values. Supratrochlear spur height was significantly increased in types B and D compared with types A and C.

TTTG distance and sTTTG distance both showed significant increases in higher‐grade dysplasia. Type D differed significantly from types A, B, and C for TTTG distance, whereas types B and D showed higher sTTTG values than types A and C. In contrast, TT‐PCL distance did not show significant intergroup differences. FT rotation increased with dysplasia severity, with type D differing significantly from lower‐grade subtypes and type A exhibiting the lowest values.

## DISCUSSION

The most important finding of this study is that high‐grade TD (Dejour grades B and D) exhibits a stronger association with more pronounced abnormalities in PF parameters than low‐grade TD in patients with patellar instability. Notably, Dejour types B and D are not only linked by the common feature of the supratrochlear spur but also exhibit similar PF abnormalities. These alterations are more pronounced than those seen in types A or C, suggesting that predominantly types B and D should not be considered in isolation but rather as part of a complex PF dysplasia. Therefore, comprehensive diagnostics are necessary to fully assess risk factors for PF instability in patients presenting with TD.

In accordance with the sex and gender equity in research recommendations, sex‐stratified analyses were performed. Significant sex‐related differences were observed for patellar width, patellar thickness, patellar tilt, TT‐PCL distance, and sTTTG distance. Although no significant differences in the distribution of Dejour TD types were identified between female and male patients, the observed differences in several anatomical parameters suggest that biological sex may contribute to specific aspects of PF morphology. Dedicated studies specifically designed and powered to investigate sex‐specific anatomical differences are warranted.

Our findings are particularly significant because previous studies have analyzed various anatomical factors and their individual prevalence but have rarely examined the co‐occurrence of these factors within individual patients.^33^, ^34^ Although relations between TD and factors such as rotational alignment, TTTG distance, or patellar tilt have been described, studies systematically comparing all 4 Dejour types across a broad range of PF anatomical parameters remain limited.^6^, ^13^, ^15^, ^16^, ^35^, ^36^ Our analysis expands on this body of literature by comprehensively evaluating multiple PF risk factors, including TTTG, sTTTG, femoral torsion, spur height, PTI, sulcus depth, trochlear sulcus angle, and Merchant's congruence angle, across all Dejour types. In doing so, we show that Dejour types B and D share a distinct pattern of associated abnormalities and consistently accumulate more severe PF alterations than types A and C. These findings support the concept that types B and D represent a distinct anatomical and biomechanical entity within the spectrum of TD.

Our results align with previous research highlighting the interplay between TD and other key parameters such as patellar tilt, patella alta, TTTG, and patellar lateralization.^5^, ^36^ An important study by Imhoff et al. showed that nearly all PF parameters correlate to some extent, emphasizing the need to consider TD as part of a broader pathological framework.^16^

Higher values for trochlear sulcus angle and supratrochlear spur height and lower values for sulcus depth, as well as the increased prevalence of heightened FT rotation, PTI, patellar tilt, TTTG, sTTTG, and Merchant's congruence angle in patients with Dejour types B and D seen in the current study, suggest that both Dejour types may be anatomically closely related regarding PF instability. Consequently, it can be proposed to consider Dejour types B and D separately instead of grouping types B, C, and D together as high‐grade TD, as proposed by Lippacher et al.^37^ Interestingly, this concept has been adopted by Dejour et al.,^38^ who recently updated their classification to define a TD type 3 characterized by a spur height >5 mm, which primarily includes types B and D. Based on the associations observed in the current study, we agree that this new classification better captures the anatomical entity of TD with its implications on PF instability.

The pronounced presence of additional risk factors for PF instability such as increased TTTG and joint incongruence in Dejour types B and D may be explained by the particularly disturbed trochlear morphology and the altered biomechanics associated with these dysplasia types.^16^, ^39^ According to the CDI measurement, patella alta is also frequently observed; however, PTI‐based assessment does not confirm patella alta in these cases and, in contrast, may even show elevated PTI values in high‐grade TD.^40^, ^41^ This discrepancy may be explained by the fact that in TD, the proximal trochlea is often flattened, widened, or even convex. As a result, the proximal patella may appear to have greater overlap with the trochlea. However, the PTI measures only the sagittal overlap between patellar and trochlear cartilage, without accounting for the quality or depth of bony containment.^42^ In knees with TD, this can lead to a “false” elevation of the PTI, where overlap is present but does not reflect true functional engagement or stability of the PF joint.^42^ In our cohort, we did not find a significant association between TD and the CDI. Patellar height has been reported to be associated with TD in several studies.^11^ However, the severity of TD does not appear to correlate with patellar height; in other words, high‐grade TD is not necessarily associated with increased patellar height. One possible explanation is that the previous classification system does not fully capture the true severity of TD. This limitation may be one of the factors motivating the updated classification proposed by Dejour, which our study aims to further address.

Van Haver et al. showed that Dejour types B and D cause the most severe alterations in PF biomechanics including increased lateral patellar translation and tilt, higher contact pressures, and reduced contact area.^3^ These biomechanical changes compromise patellar tracking and may lead to altered PF engagement and congruence.

Furthermore, the current study did not confirm previous findings, suggesting an association between the severity of trochlear and patellar dysplasia,^43^ because no differences in patellar angle, thickness, or width were observed across Dejour types. However, the patellar angle in this cohort was consistent with that reported in a prior study comparing patients with and without TD, showing that both TD and patellar dysplasia are associated with each other.^6^ Consequently, it is believed that a reciprocal developmental mechanism, in which chronic instability leads to maladaptive remodeling of both joint partners, contributes to PF dysplasia.^43^ During growth, the shape of the trochlea and patella is influenced by mutual mechanical loading and adaptation. A dysplastic, flat, or convex trochlea may fail to guide the patella properly, resulting in abnormal loading and secondary changes in patellar morphology.^44^ Conversely, a dysplastic patella may impair trochlear development. This interdependence reinforces the pathological biomechanics of the PF joint, contributing to maltracking, lateral translation, and instability.^3^, ^4^, ^43^

Taken together, these findings suggest that patients with Dejour types B and D are more likely to present with multiple anatomical risk factors for PF instability. Clinically, this means that these patients face a higher risk of recurrent patellar dislocations and poorer functional outcomes if these combined factors are not appropriately addressed during diagnosis and treatment.^45^

The results of this study emphasize the necessity of comprehensive anatomical assessment in patients with patellar instability, particularly in those with Dejour types B and D. In such cases, isolated soft tissue procedures such as MPFL reconstruction may not be sufficient.^46^ Instead, additional surgical interventions, such as trochleoplasty, tibial tubercle osteotomy, or other patellar realignment procedures, including femoral derotational osteotomy or correction of a valgus limb alignment when indicated, should be considered to ensure long‐term stability.

### Limitations

This study is not without limitations. First, the retrospective design and potential selection bias inherent in our patient population may have influenced the results. Second, reliable data regarding the number of patellar dislocations was not available. Third, this study did not assess patient‐reported outcome measures, which limit the correlation of anatomical findings with clinical outcomes. Additionally, this study solely reports associations between TD and other PF anatomical parameters and does not prove a causative relation. Furthermore, inter‐ and intraobserver reliability were not assessed. Therefore, the consistency and reproducibility of the measurements could not be determined, and observer‐related variability may have influenced the results. Finally, because no a priori sample size calculation was performed, the study may be underpowered, increasing the risk of a type II (*β*) error and the possibility that clinically relevant differences were not detected.

## CONCLUSIONS

Patients with Dejour type B and D TD had significantly increased values in other PF instability risk factors than those presenting with type A and C dysplasia.

## DECLARATION OF GENERATIVE AI AND AI‐ASSISTED TECHNOLOGIES IN THE WRITING PROCESS

During the preparation of this work, the authors used ChatGPT and openevidence.com to support translation, ensure grammatical correctness, and assist in source identification. After using this tool, the authors reviewed and edited the content as needed and take full responsibility for the content of the publication.

## DISCLOSURES

The authors (M.H., B.C., L.V., J.A., S.F.F.) declare that they have no known competing financial interests or personal relationships that could have appeared to influence the work reported in this paper.

### FUNDING

Open access publishing facilitated by Universitat Zurich, as part of the Wiley ‐ Universitat Zurich agreement via the Consortium Of Swiss Academic Libraries.
